# miR-206-3p Inhibits 3T3-L1 Cell Adipogenesis via the c-Met/PI3K/Akt Pathway

**DOI:** 10.3390/ijms18071510

**Published:** 2017-07-14

**Authors:** Renqiao Tang, Feifei Ma, Wei Li, Shengrong Ouyang, Zhuo Liu, Jianxin Wu

**Affiliations:** 1 Graduate School of Peking Union Medical College, NO. 9, Dongdansantiao, Dongcheng District, Beijing 100730, China; tmanbridge@163.com (R.T.); libuwei2011@163.com (W.L.); 2 Department of Biochemistry, Capital Institute of Pediatrics, NO. 2, Yabao Road, Chaoyang District, Beijing 100020, China; mafei0817@163.com (F.M.); cipbiochem@163.com (S.O.); liuzhuozhuo2005@163.com (Z.L.)

**Keywords:** miR-206-3p, c-Met, Akt signaling, adipogenesis

## Abstract

MicroRNAs (miRNAs) are important post-transcriptional regulators during adipocyte adipogenesis. MiR-206-3p, a tissue-specific miRNA, is absent in white adipocytes. In this study, we examined the roles of mmu-miR-206-3p in the adipogenic differentiation of 3T3-L1 preadipocytes. The miR-206-3p expression has shown an apparent decreasing trend after induction, and sustained low expression throughout the differentiation of 3T3-L1 cells. miR-206-3p blocked the adipogenic differentiation of 3T3-L1 cells by attenuating c-Met expression; the inhibition effect of miR-206 to the adipogenic differentiation can be counteracted by restoring c-Met expression. In addition, miR-206-3p decreased the phosphorylation of Akt, which is the downstream effector of c-Met in the PI3K/Akt signaling pathway. These data indicate that miR-206-3p inhibits adipocyte adipogenesis through silencing c-Met and subsequently inactivating the PI3K/Akt signaling pathway.

## 1. Introduction

MicroRNAs (miRNAs) are endogenous noncoding RNAs which negatively regulate gene expression by targeted binding to the 3′ untranslated region (3′UTR) of mRNAs at the post-transcriptional level [1,2]. miRNAs play roles in numerous biological processes including embryonic development, organ morphology, tumorigenesis, and cell differentiation [3,4,5,6,7].

Adipocyte differentiation is a dynamic and complex process, and many miRNAs involved in adipogenic differentiation have been identified [8,9]. Recently, miRNA microarray analysis showed that miR-206 was downregulated remarkably and sustained low expression throughout adipogenesis under induction culture condition [10]. miR-206, a member of the so-called myomiR family (i.e., miR-1, miR-133, and miR-206), is largely acknowledged as a specific, positive regulator of skeletal muscle differentiation [11]. miR-206 is absent in white adipocytes but specifically expressed in brown adipocytes [12]. miR-206 has been well studied in various tumor cells, and downregulated miR-206 is observed in different types of cancers [13,14,15]. miR-206 can promote apoptosis, induce cell cycle arrest, and inhibit cell migration and invasion in various cancers. miR-206 played roles in various functions by targeting c-Met and its downstream PI3K/Akt pathway [16], which is essential for adipocyte differentiation [17,18,19]. All the above clues suggest that miR-206 might play a specific role in adipogenesis.

In the current study, we attempted to uncover the role of miR-206 in adipogenic differentiation. We confirmed that miR-206 expression was downregulated throughout 3T3-L1 adipogenesis, and that the ectopic introduction of miR-206 resulted in the decreased expression of specific adipogenic markers. Further experiments validated that the inhibition effect of miR-206 can be counteracted by c-Met overexpression, accompanied with enhanced phosphorylation of Akt, which is the downstream effector of c-Met in the PI3K/Akt signaling pathway. These results suggest the potential use of miR-206 in the therapy of obesity.

## 2. Results

### 2.1. Expression Level of miR-206 during the Differentiation of 3T3-L1 Cells

**Figure 1:**
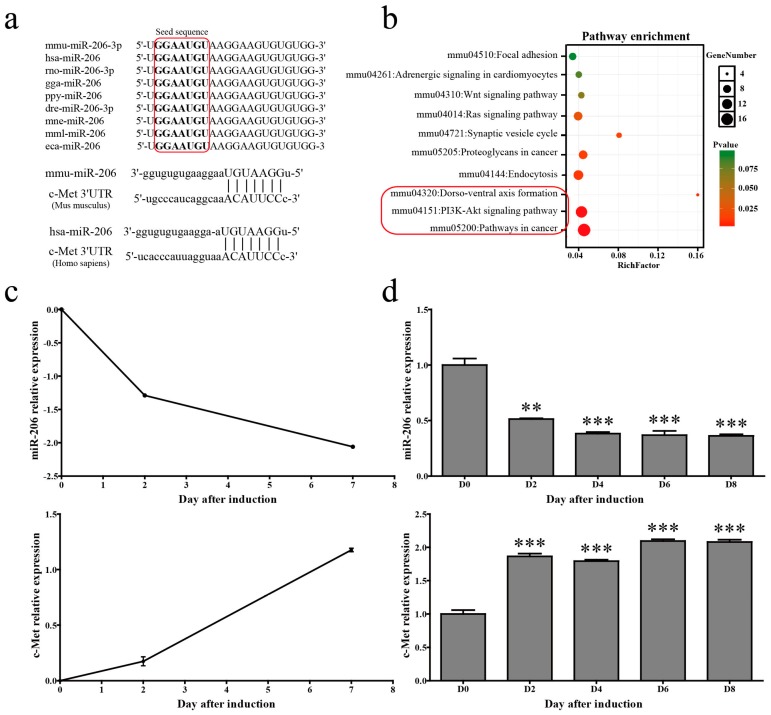
miR-206-3p was downregulated during 3T3-L1 differentiation. (**a**) A mature miR-206-3p sequence is conserved among different species, and sequence alignment between miR-206 and c-Met mRNA 3′UTR of mouse and human is observed. The sequences in the red frame are miR-206 seed sequences of different species; (**b**) KEGG pathway analysis for predicted target genes of mmu-miR-206-3p. The pathways in the red frame are the top 3 most significant enrichment pathways, which include PI3K/Akt pathway; (**c**) miR-206 and c-Met expression levels were detected in microarray profile; and (**d**) verified by qPCR during 3T3-L1 differentiation. U6 snRNA was used as a reference gene. Data are shown as means ± SD, ** *p* < 0.01, *** *p* < 0.001.

A mature miR-139-5p sequence is highly conserved in many mammals including mice, humans, and chickens. The alignment of mouse and human c-met 3′UTRs with the miR-206 “seed” region revealed a high degree of evolutionary conservation (Figure 1a). Pathway analysis for the predicted target genes of mmu-miR-206-3p revealed that the main functions of mmu-miR-206-3p were related to cancer and the PI3K/Akt pathway (Figure 1b). Time course analysis uncovered that miR-206 showed a decreased trend of expression in microarray assays during 3T3-L1 cells adipogenic differentiation (Figure 1c, up). 3T3-L1 cells were collected at different time points (i.e., 0, 2, 4, 6, and 8 days) after 1-methyl-3-isobutylxanthine, dexamethasone, and insulin (MDI) stimulation to confirm the changes of miR-206 during adipogenic differentiation. Stem-loop quantitative polymerase chain reaction (qPCR) results show that miR-206 gradually decreased after MDI stimulation, and were maintained at low level throughout adipogenesis (Figure 1d, up). Meanwhile, c-Met, the target gene of miR-206, has shown sustained increasing trends both in microarray profile (Figure 1c, bottom) and qPCR verification (Figure 1d, bottom). These results suggest that miR-206 is likely associated with adipogenesis.

### 2.2. Effect of miR-206 on the Adipogenic Differentiation of 3T3-L1 Cells

**Figure 2:**
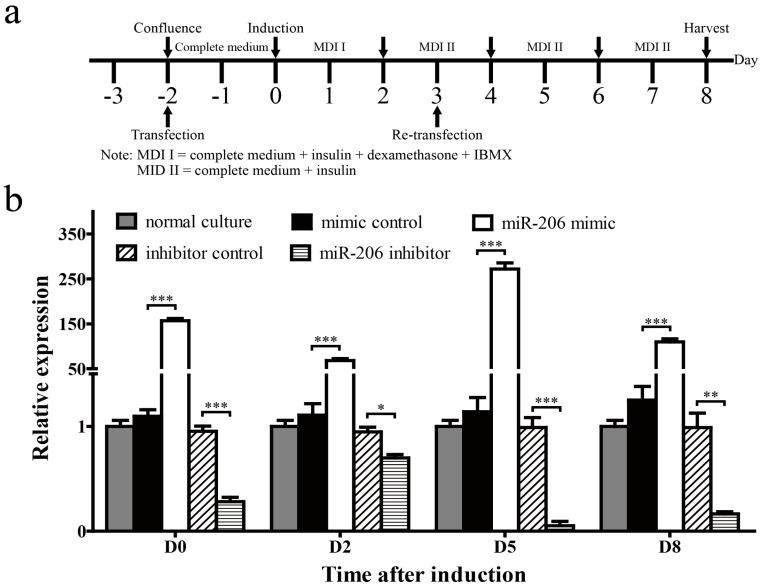
Examination for the transfection efficiency and stability of miR-206-3p during 3T3-L1 differentiation. (**a**) Schematic diagram of the treatment schedule for cell induction and transfection during 3T3-L1 differentiation; (**b**) Expression of miR-206-3p was detected on D0, D2, D5, and D8 after MDI induction. U6 snRNA was used as a reference gene. Data are shown as means ± SD, * *p* < 0.05, ** *p* < 0.01, *** *p* < 0.001.

3T3-L1 preadipocytes were transfected with miR-206 mimic or inhibitor to determine the role of miR-206 in adipogenic differentiation (Figure 2a). First, we collected cells on days 0, 2, 5 and 8 (D0, D2, D5, D8, respectively) after MDI induction to determine the transfection efficiency and stability of miR-206. Mature miR-206 increased 156-fold in 3T3-L1 cells on D0 with miR-206 mimic transfection and maintained at a high level until harvest. By contrast, the mature miR-206 was reduced to one-fourth on D0 with miR-206 inhibitor transfection (Figure 2b). These results show that miR-206 mimics and inhibitors are able to perform in our subsequent research.

**Figure 3:**
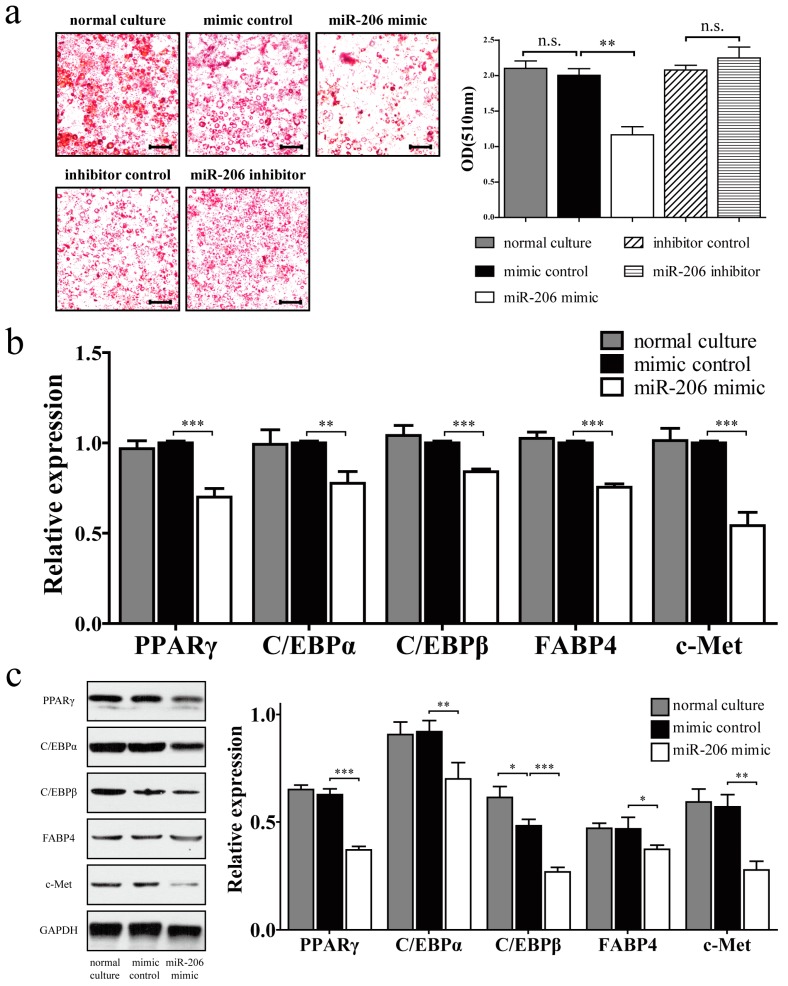
miR-206 inhibited the adipogenic differentiation of 3T3-L1 preadipocytes. (**a**, **left**) 3T3-L1 preadipocytes were transfected with miR-206 mimic or inhibitor, cells were fixed and stained with Oil Red O on D8 after MDI stimulation. Scale bar, 100 μm; (**a**, **right**) Lipid accumulation was quantified by extracting Oil Red O, and the absorbance was measured at 510 nm wavelength; (**b**) The mRNA expression of PPARγ, C/EBPα, C/EBPβ, FABP4, and c-Met on D8 after MDI stimulation were measured by RT-qPCR; (**c**) The protein levels of PPARγ, C/EBPα, C/EBPβ, FABP4, and c-Met on D8 after MDI stimulation were detected by Western blotting (**left**) and quantified by densitometry analysis (**right**). GAPDH protein levels served as a loading control. Data are shown as means ± SD, * *p* < 0.05, ** *p* <0.01, *** *p* < 0.001, n.s. stands for not significant.

miR-206 inhibited the adipogenic differentiation of 3T3-L1 preadipocytes, as demonstrated by a reduction of Oil Red O staining (Figure 3a, left). Meanwhile, the negative control showed no obvious difference during adipogenic differentiation. Quantitative analysis also revealed statistically significant inhibition of intracellular lipids accumulation. However, no obvious promotion effect was observed after miR-206 inhibitor treatment (Figure 3a, right). This observation may be due to the scarce miR-206 in differentiated adipocytes, due to which the inhibitor cannot work effectively. Several markers of adipogenic differentiation were detected by real-time qPCR (RT-qPCR) and Western blotting. In comparison with the control group, miR-206 mimic significantly downregulated the expression of PPARγ, C/EBPα, C/EBPβ, and FABP4 in mRNA and protein levels (Figure 3b,c). Collectively, these results show that miR-206 can inhibit 3T3-L1 preadipocyte differentiation. Meanwhile, the target gene c-Met was also significantly downregulated with miR-206 overexpression (Figure 3b,c).

### 2.3. c-Met Restores the Inhibition Effect of miR-206

c-Met was shown to be a target of miR-206, and its principal role needs to be evaluated during 3T3-L1 preadipocyte differentiation. Knockdown of c-Met can reduce mouse fat weight, and downregulate expression levels of PPARγ and lipoprotein lipase (LPL) in fat pads [20]. Thus, we examined whether constitutive c-Met expression can counteract the repression effect of miR-206 on adipogenesis.

**Figure 4:**
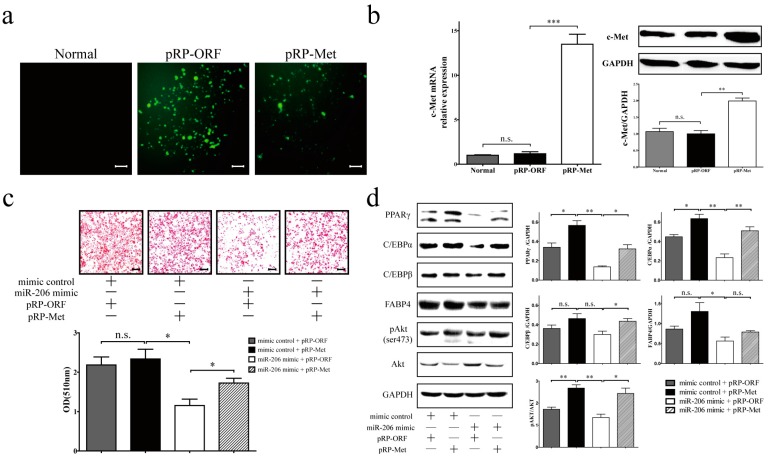
c-Met overexpression can overcome the inhibition effect of miR-206 in 3T3-L1 cells. (**a**) The overexpression of c-Met was through the transfection of regular plasmid gene expression vector (pRP-Met) into 3T3-L1 preadipocytes; the expression of enhanced green fluorescent protein (EGFP) was monitored in the transduced cells byoptical microscopy. Scale bar, 100 μm; (**b**) c-Met expression was detected on the mRNA level (**b**, **left**) and protein level (**b**, **right**); (**c**) 3T3-L1 preadipocytes were co-transfected with miR-206 mimic and pRP-Met, cells were fixed and with Oil Red O stained lipid on D8 after MDI stimulation (**up**), and quantitative analysis with Oil Red O (**bottom**). Scale bar, 100 μm; (**d**, **left**) The protein levels of PPARγ, C/EBPα, C/EBPβ, FABP4, c-Met, Akt, and pAkt on D8 after MDI stimulation were detected by Western blotting and (**right**) quantified by densitometry analysis. GAPDH protein levels served as a loading control. Data are shown as means ± SD, * *p* < 0.05, ** *p* < 0.01, *** *p* < 0.001, n.s. stands for not significant.

The overexpression of c-Met by transfecting plasma vector pRP-Met (Figure 4a), as evidenced by the raised expression of c-Met mRNA and protein (Figure 4b), resulted in a slight increase of differentiation efficiency in 3T3-L1 cells, though this difference did not reach statistical significance (Figure 4c, mimic control + pRP-Met). However, c-Met overexpression can enhance 3T3-L1 cell differentiation effectively in the presence of transfected miR-206 mimic (Figure 4c, miR-206 mimic + pRP-Met). As expected, the presence of miR-206 mimic with control empty plasmid (pRP-ORF) in 3T3-L1 preadipocytes led to the suppression of 3T3-L1 cell differentiation (Figure 4c, miR-206 mimic + pRP-ORF). Furthermore, the decrease in adipogenic marker gene expression (PPARγ, C/EBPα, and C/EBPβ) as a result of miR-206 was attenuated by the co-expression of c-Met (Figure 4d). However, FABP4 did not reach statistical significance on the protein level (Figure 4d). Overall, c-Met overexpression can restore adipocyte differentiation and overcome the inhibition effect of miR-206.

### 2.4. miR-206 Inactivates the c-Met/PI3k/Akt Pathway

**Figure 5:**
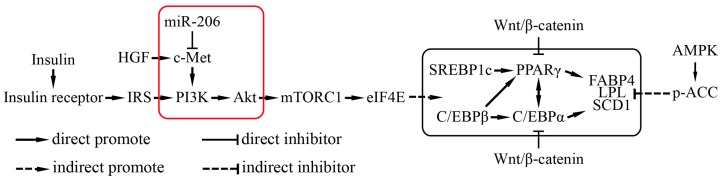
A schematic of the miR-206 regulatory mechanism during adipogenesis. The red frame is the results of our study and their position in the regulation of adipogenesis.

Hepatocyte growth factor (HGF)/c-Met (also named as hepatocyte growth factor receptor, HGFR) signals via a number of intracellular signaling mechanisms, including PI3k/Akt, Ras/MAPK, and JAK/STAT pathways, to increase scattering/motility, invasion, proliferation, survival, and morphogenesis [21,22]. This study shows that, after transfection with c-Met, the phosphorylation of Akt was notably augmented (Figure 4d). By contrast, the phosphorylation of Akt was decreased when transfected with miR-206 mimic (Figure 4d). Collectively, miR-206 inhibited adipocyte differentiation through decreasing c-Met protein translation and further decreasing the phosphorylation of Akt level, the latter being activated the transcription of genes that enhanced adipocyte differentiation (Figure 5).

## 3. Discussion

Obesity is featured with increases in both adipocyte size and number. Mature adipocytes arise from preadipocytes or progenitor cells, which can differentiate into adipocytes in response to a certain signal. Adipocyte differentiation is a complex process with the precise regulation of several levels. Transcription factors, such as PPARγ and C/EBPα, and signaling pathways, such as PI3K/Akt, insulin, and Wnt/β-catenin signaling pathways, have been extensively studied [23,24]. In addition, an increasing number of miRNAs have been identified to be involved in adipogenesis.

In this study, we demonstrated that miR-206 can inhibit 3T3-L1 preadipocyte differentiation via the c-Met/PI3K/Akt signaling pathways. miR-206, which is largely acknowledged as a specific, positive regulator of skeletal muscle differentiation, can promote apoptosis, induce cell cycle arrest, and inhibit cell migration and invasion in various cancers [13,14,15,25,26,27,28,29,30,31,32]. miR-206 can inhibit lipogenesis by suppressing the expression and activity of liver X receptor α (LXRα) in hepatocytes [33]. c-Met, which is a major target gene of miR-206, is widely recognized as an important upstream regulator of the PI3K/Akt pathway. c-Met proto-oncogene (regarded as HGFR) is a receptor tyrosine kinase that binds to HGF ligand. HGF is a multifunctional cytokine that binds to c-Met to regulate many physiological processes including proliferation, scattering, morphogenesis, survival, migration, and invasion in a variety of systems [34]. HGF can promote glucose uptake through the PI3K/Glut4 pathway in 3T3-L1 adipocytes [35], and knockdown of c-Met by siRNA can reduce mouse fat pad weight, along with low expression levels of PPARγ and LPL in fat pads [20]. In this study, c-Met overexpression can increase Akt phosphorylation and enhance adipocyte differentiation. Therefore, the overexpression of c-Met can counteract the inhibition effect of miR-206 on adipogenesis.

## 4. Materials and Methods

### 4.1. Silico Experiment

In the present work, a time course analysis was performed based on the transcription profile of a microarray data series (GSE20696) and miRNA expression profile (GSE20698) using the Short Time-Series Expression Miner software. GSE20696 and GSE20698 are expression profiles of 3T3-L1 adipogenesis, first published by Mikkelsen et al. [10] from Broad Institute, MA, USA. miRNA targets were predicted by miRecords (available online: http://c1.accurascience.com/miRecords/) [36]. The functional annotation of relevant gene clusters was performed using the DAVID online tool (available online: http://david.abcc.ncifcrf.gov/) [37].

### 4.2. Cell Culture and Differentiation

Mouse 3T3-L1 cell lines were obtained from the American Type Culture Collection. The cell lines were cultured in Dulbecco modified Eagle medium (DMEM, Gibco™, Thermo Fisher Scientific, Waltham, MA, USA) with 10% fetal bovine serum (FBS, Gibco™, Thermo Fisher Scientific) at 37 °C in a 5% CO_2_ incubator. Adipogenic differentiation was carried out according to the classical MDI (1-methyl-3-isobutylxanthine, dexamethasone and insulin) cocktail. 3T3-L1 preadipocytes were grown to confluence. Two days later (designated as D0), 3T3-L1 preadipocytes were stimulated for two days in differentiation medium: DMEM containing 10% FBS and MDI (0.5 mm 3-isobutyl-1-methylxanthine, 1 μm dexamethasone, and 5 μg/mL insulin). Two days later, the medium was changed to DMEM containing 10% FBS and 5 μg/mL insulin, and the medium was refreshed every two to three days until harvest.

### 4.3. Oil Red O Staining

3T3-L1 cells were washed with PBS and fixed with 4% paraformaldehyde for 1 h. After washing thrice, the cells were stained with 60% saturated Oil Red O at 37 °C for 30 min, washed twice, and then photographed. The intracellular absorbed Oil Red O was extracted in pure isopropanol, and absorbance was measured at 510 nm wavelength with a spectrophotometer (Varioskan Flash, Thermo Scientific), which was an indicator of intracellular lipid accumulation by quantification.

### 4.4. Cell Transfection

miRNA mimic and inhibitor were synthesized by RiboBio Co., Ltd. (Guangzhou, China); c-Met overexpression plasmid vector [38] (termed pRP-Met) or blank vector (termed pRP-ORF) were purchased from Cyagen Biosciences Inc. (Santa Clara, CA, USA). All the oligonucleotide and plasmid transfections were performed using Lipofectamine 2000 (Invitrogen™, Thermo Fisher Scientific) according to the manufacturer’s instruction. For adipocyte differentiation, the 3T3-L1 cells were transfected with 50 nm miRNA mimics, 100 nm miRNA inhibitor, or 8 μg/mL plasmid. To increase the transfection efficiency, transfection was repeated twice every five days (on D2 and D3, Figure 2a).

### 4.5. Real-Time Quantitative Polymerase Chain Reaction

Total RNA was extracted from cells using Trizol reagent (Invitrogen™, Thermo Fisher Scientific) according to the manufacturer’s instructions. After quantification, cDNA was obtained using the Reverse Transcription System (Promega, Corporation, Fitchburg, MA, USA), with oligo (dT) primer for mRNA and stem-loop reverse transcription primer for miRNA. The mRNA expression level of genes was examined by RT-qPCR using UltraSYBR Mixture (CoWin Biosciences, Beijing, China) with the following pairs of primers: PPARγ forward, 5′-TGTGGGGATAAAGCATCAGGC-3′, and reverse 5′-CCGGCAGTTAAGATCACACCTAT-3′; C/EBPα forward, 5′-GCTGGAGTTGACCAGTGACA-3′, and reverse 5′-AAACCATCCTCTGGGTCTCC-3′; C/EBPβ forward, 5′-GCAAGAGCCGCGACAAG-3′, and reverse 5′-GGCTCGGGCAGCTGCTT-3′; FABP4 forward, 5′-ACACCGAGATTTCCTTCAAACTG-3′, and reverse 5′-CCATCTAGGGTTATGATGCTCTTCA-3′; c-Met forward, 5′-ACCAAGTGCTCCTGACATCC-3′, and reverse 5′-GTGAGGTGTGCTGTTCGAGA-3′;GAPDH forward, 5′-AAGAAGGTGGTGAAGCAG-3′, and reverse 5′-GAAGGTGGAAGAGTGGGAGT-3′; U6 snRNA forward, 5′-CTCGCTTCGGCAGCACA-3′, and reverse 5′-AACGCTTCACGAATTTGCGT-3′; mmu-miR-206-3p forward, 5′-ACACTCCAGCTGGGTGGAATGTAAGGAAGTGT-3′, and reverse 5′-TGGTGTCGTGGAGTCG-3′. Relative quantification (2^−ΔΔ*C*t^) method was used to analyze the data, GAPDH mRNA was used as reference for mRNA quantification, and U6 snRNA was used for miRNA.

### 4.6. Western Blot

Cells were lysed using cell lysis buffer (RIPA, Cell Signaling Technology Inc. (CST), Danvers, MA, USA) with protease inhibitor. Total protein from samples was electrophoresed on SDS-PAGE and transferred to polyvinylidene fluoride (PVDF) membrane. The membranes were then blocked with Tris buffered saline with Tween 20 (TBS-T, 10 mm Tris-HCl, 150 mm NaCl, and 0.1% Tween 20) containing 5% skim milk for 2 h and incubated at 4 °C overnight with primary antibodies. Antibodies against c-Met (Abcam, Cambridge, UK), anti-PPARγ (CST, Danvers, MA, USA), anti-C/EBPα (CST), anti-C/EBPβ (CST), anti-FABP4 (CST), anti-GAPDH (CST), anti-Akt (CST), and anti-pAkt (CST) were used. The secondary antibodies conjugated to horseradish peroxidase were then added and incubated for 1 h. Enhanced chemiluminescence reagent was used to visualize blots (CoWin Biosciences, Beijing, China). The blot intensity was photographed and quantified by chemiluminescent Imaging and Analysis System (MiniChemi, Beijing, China).

### 4.7. Statistical Analysis

All quantitative results are presented as means ± standard deviation (SD) unless otherwise stated. Statistical significance among multiple groups was analyzed by one-way ANOVA, and between two groups by independent *t*-test. A value of *p* < 0.05 (significant) or *p* < 0.01 (extremely significant) was considered to be statistically significant.

## 5. Conclusions

In conclusion, our study has proven that the miR-206 suppressed adipocyte differentiation by inhibiting c-Met at the post-transcriptional level and further reduced the activity of the downstream PI3K/Akt signaling pathway. The inhibition effect of miR-206 to the adipogenic differentiation can be counteracted by restoring c-Met expression. Therefore, miR-206 and its target genes may be novel targets for reducing excess fat.

## Abbreviations

PPARγ	Peroxisome proliferator-activated receptor γ
C/EBPα	CCAAT/enhancer binding protein α
C/EBPβ	CCAAT/enhancer binding proteinβ
FABP4	Fatty acid binding protein4
PI3K	Phosphatidylinositol 3-kinase
GAPDH	Glyceraldehyde-3-phosphate dehydrogenase
HGF	Hepatocyte growth factor
HGFR	Hepatocyte growth factor receptor
